# Numerical Simulation Study of the Mechanical Behaviour of 1D and 2D Germanium Carbide and Tin Carbide Nanostructures

**DOI:** 10.3390/ma16155484

**Published:** 2023-08-05

**Authors:** José V. Fernandes, André F. G. Pereira, Jorge M. Antunes, Bruno M. Chaparro, Nataliya A. Sakharova

**Affiliations:** 1 Centre for Mechanical Engineering, Materials and Processes (CEMMPRE), Advanced Production and Intelligent Systems, Associated Laboratory (ARISE), Department of Mechanical Engineering, University of Coimbra, Rua Luís Reis Santos, Pinhal de Marrocos, 3030-788 Coimbra, Portugal; valdemar.fernandes@dem.uc.pt (J.V.F.); andre.pereira@uc.pt (A.F.G.P.); jorge.antunes@dem.uc.pt (J.M.A.); 2 Abrantes High School of Technology, Polytechnic Institute of Tomar, Quinta do Contador, Estrada da Serra, 2300-313 Tomar, Portugal; bruno.chaparro@ipt.pt

**Keywords:** germanium carbide, tin carbide, nanosheets, nanotubes, elastic moduli, Poisson’s ratio, force field constants, numerical simulation

## Abstract

One-dimensional (nanotubes) and two-dimensional (nanosheets) germanium carbide (GeC) and tin carbide (SnC) structures have been predicted and studied only theoretically. Understanding their mechanical behaviour is crucial, considering forthcoming prospects, especially in batteries and fuel cells. Within this framework, the present study aims at the numerical evaluation of the elastic properties, surface Young’s and shear moduli and Poisson’s ratio, of GeC and SnC nanosheets and nanotubes, using a nanoscale continuum modelling approach. A robust methodology to assess the elastic constants of the GeC and SnC nanotubes without of the need for numerical simulation is proposed. The surface Young’s and shear moduli of the GeC and SnC nanotubes and nanosheets are compared with those of their three-dimensional counterparts, to take full advantage of 1D and 2D germanium carbide and tin carbide in novel devices. The obtained outcomes establish a solid basis for future explorations of the mechanical behaviour of 1D and 2D GeC and SnC nanostructures, where the scarcity of studies is evident.

## 1. Introduction

In recent times, there has been an increasing demand for one-dimensional (1D) and two-dimensional (2D) materials with a graphene-like lattice for applications in optoelectronics and energy engineering. Binary compounds constituted by carbon (C) and other elements of the 14th group of the periodic table, such as germanium (Ge) and tin (Sn), can form stable 2D graphene-like structures (monolayers) [1,2]. Hexagonal germanium carbide (GeC) monolayers have great potential for the design and fabrication of blue and ultraviolet light-emitting diodes [3,4], anti-reflection and infrared window protection coatings [3] and in photoelectronics [5]. According to Li et al. [6], the heterostructure, in which one GeC monolayer is combined with one of boron phosphide (BP), has potential for application in nanoelectronic devices due to tuned semiconductor-to-metal transition. Various 2D carbides have already been pointed out as novel efficient materials for electrodes in sodium-ion and lithium-ion batteries [7,8]. For example, it has been shown using first-principles calculations that the GeC monolayer can be considered as a prospective cathode catalyst for fuel cells and lithium–oxygen batteries [9]. Moreover, Khossossi et al. [10], employing density functional theory (DFT) calculations, proposed the GeC monolayer as a novel anode material for Li/Na-ion batteries. Rehnam et al. [8], in their recent review, suggested germanium and tin carbide and sulphites as promising anode materials for Na-ion batteries due to their structural stability and excellent electrochemical performance.

The tin carbide (SnC) monolayer has a large indirect band gap of 1.71 eV [2] or 1.5 eV [11]. Along with the theoretically envisaged possibility of making the semiconductor transform into a semimetal and the indirect-to-direct bandgap transitions by applying tensile and compressive strains to SnC monolayers [2,12], it supports a particular importance of SnC nanosheets for optoelectronics. With regard to prospects in energy storage applications, a 2D tin carbide nanosheet with a small tensile strain applied has been shown, using first-principles calculations, to be a suitable anode material for Li-ion batteries [13]. Moreover, Marcos-Viquez et al. [14], based on DFT calculations, pointed out to the capability of SnC nanosheets to act as toxic gas sensors.

Regarding the aforementioned potential applications of GeC and SnC nanosheets, most of the studies performed so far have dealt with the evaluation of their structural, electronic and optical properties. Şahin et al. [1], based on first-principles calculations, predicted stable hexagonal lattices with planar geometry for GeC and SnC monolayers and calculated their bond length, 2D hexagonal lattice constant and electronic band structure, using DFT with local density approximation (LDA). Sohbatzadeh et al. [15] investigated the structure of GeC monolayers employing ab initio calculations within DFT and determined the values of the equilibrium lattice constant and Ge–C bond length. Hoat et al. [16] showed the dynamic stability of the planar structure of the SnC monolayer and evaluated its band gap using DFT calculations with the Heyd–Scuseria–Ernzerhof hybrid functional (HSE06). Kumar et al. [17] studied an optimized structure and calculated the band-gap energy of armchair and zigzag GeC nanoribbons, employing first-principles calculations for this purpose. Yu et al. [18] performed calculations using the pseudopotential plane-wave method to investigate the structural stability and evaluate the band-gap energy of the GeC monolayer. The structural properties (bond length), electronic density and band structure of SnC nanosheets, pristine and containing point defects, were evaluated by Majidi et al. [19], who used first-principles calculations within DFT employing numerical atomic orbitals (NAO) as the basis for their simulation study. Lu et al. [2] calculated the electronic structure of GeC and SnC monolayers using first-principles calculations based on DFT and the quasiparticle GW method. Behzad and Chegel [4] investigated by DFT the electronic and optical properties of the GeC nanosheet. In their turn, Mogulkoc et al. [12] studied the electronic and optical properties of strained SnC nanosheets by combining DFT and tight-binding models. The abovementioned studies point to germanium carbide and tin carbide monolayers as semiconductors with a large indirect band gap, which, together with the knowledge of their structural stability, confirms that GeC and SnC nanosheets are suitable components for novel applications in nanoelectronics. Studies regarding mechanical properties are less common in the literature. For example, Peng et al. [3] and Sohbatzadeh et al. [15] calculated the elastic constants of GeC nanosheets, in both cases using ab initio calculations within DFT. Taking into account that the band structure and optical properties of GeC and SnC nanosheets can be adjusted by modulating the applied strain [2,4], the knowledge of their mechanical behaviour is indispensable to the accurate design and manufacturing of the upcoming electronic and optical nanodevices and to contribute to strain engineering needs.

As far as tube-shaped (1D) GeC and SnC nanostructures are concerned, among the prospective applications envisaged for the respective nanotubes (NTs) are photocatalysis materials [20], fuel cell constituents [21], light microwave absorbers [22], nanoelectronic circuits [23], nanodevice construction [24], strain engineering [23,25] and molecular electronics [25,26]. To the best of our knowledge, neither germanium carbide nanotubes (GeCNTs) nor tin carbide nanotubes (SnCNTs) have been synthesized, although NTs of both compounds have been predicted theoretically [23,25,26,27]. Rathi and Ray [25] used hybrid density functional theory with the Hartree–Fock methodology to study the geometric and electronic structure of armchair GeCNTs. Baei et al. [23] investigated the effect of an applied electric field on the structural stability, and the electrical and electronic response of the (6, 0) zigzag GeCNT, employing DFT calculations. The projector-augmented-wave potential approach within DFT was used by Wang et al. [24] in their ab initio simulation study on the electronic and magnetic properties of the (8, 8) armchair GeCNT filled with iron nanowire. The stability and band structures of double-walled armchair GeCNTs were studied by Song and Henry [27], resorting to first-principles calculations based on DFT. Samanta and Das [26] calculated the band structures of (4, 0) to (6, 0) zigzag SnCNTs, using a combined method of DFT and non-equilibrium Green’s function. As far as we know, results about the mechanical properties of GeCNTs and SnCNTs are not available in the literature up to now.

In spite of the important potential applications envisioned for 1D and 2D nanostructures of GeC and SnC, there is a noticeable insufficiency regarding the understanding of their mechanical properties. Accurate knowledge of the mechanical behaviour of GeC and SnC nanostructures is helpful for the correct design, performance improvement and guarantee of robustness of devices for electronic, optical and energy storage needs. Against this background, the present study consists of a systematic evaluation of the elastic properties (surface Young’s and shear moduli, and Poisson’s ratio) of germanium carbide and tin carbide nanosheets (GeCNSs and SnCNSs) and single-walled germanium carbide and tin carbide nanotubes (SWGeCNTs and SnCNTs), using finite element (FE) analysis. The two sets of force field constants, necessary to provide input parameters for modelling and numerical simulation of 1D and 2D GeC and SnC nanostructures, were computed by two different methods. The influence of the input data set on the elastic properties of GeCNSs and SnCNSs, and SWGeCNTs and SWSnCNTs was studied.

## 2. Materials and Methods

### 2.1. Atomic Structure of Germanium Carbide and Tin Carbide Nanosheets and Nanotubes

**Figure 1:**
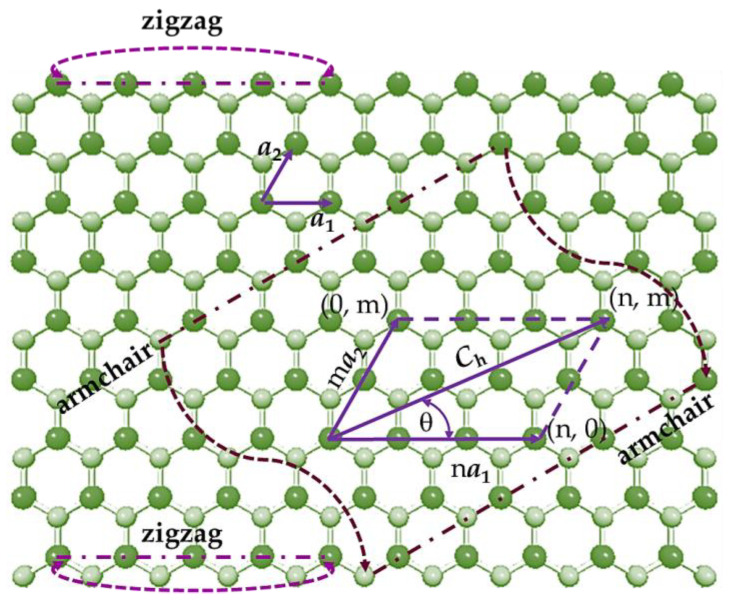
SnCNS with definitions of the chiral vector, **C_h_**, chiral angle, θ, and the scheme for rolling up armchair and zigzag nanotubes. Sn atoms are depicted in bright green; C atoms in pale green.

Both germanium carbide and tin carbide sheets have a hexagonal lattice, where the Ge (Sn) and C atoms form a honeycomb structure with planar geometry [1], as shown in Figure 1 for the case of the SnC nanosheet. The hexagonal atomic arrangement of GeCNS and SnCNS is characterized by the chiral vector, **C_h_**, and the chiral angle, θ, defined by the follow expressions, respectively:

(1) $$C_{h}=na_{1}+ma_{2}$$

(2) $$\theta =\sin^{-1}\frac{\sqrt[{}]{3}}{2}\frac{m}{\sqrt[{}]{n^{2}+\mathrm{nm}+m^{2}}},$$

where $a_{1}$ and $a_{2}$ are the unit vectors of the GeC and SnC honeycomb lattices; n and m are the chiral indices, having both integer values. The unit vector length a is expressed by $a\;=a_{A14-C}\sqrt{3}$, where $a_{A14-C}$ is the bond length in the equilibrium state, equal to 0.186 nm and 0.205 nm for Ge–C and Sn–C interatomic bonds, respectively [1].

Single-walled GeCNT and SnCNT can be understood as rolled-up GeC and SnC sheets, respectively, with the chiral angle, θ, in the range 0° ≤ θ ≤ 30°. Consequently, based on the value of θ, three main symmetry groups of NTs can be specified: zigzag (n, 0) configuration, for θ = 0° (m = 0); armchair (n, n) configuration, for θ = 30° (n = m); chiral (n, m) configuration, for 0° < θ < 30° (n ≠ m ≠ 0). The two-edged configurations, (n, 0) zigzag and (n, n) armchair (see Figure 1) are known as non-chiral NTs. The geometric characteristic of the SWGeCNTs and SWSnCNTs is the nanotube diameter, $D_{n}$, defined by the following expression:

(3) $$D_{n}=\frac{a_{A14-C}\sqrt{3\left(n^{2}+\mathrm{nm}+m^{2}\right)}}{\pi },$$

where $a_{A14-C}$ is the equilibrium bond length for GeC and SnC, and n and m are the chiral indices.

**Figure 2:**
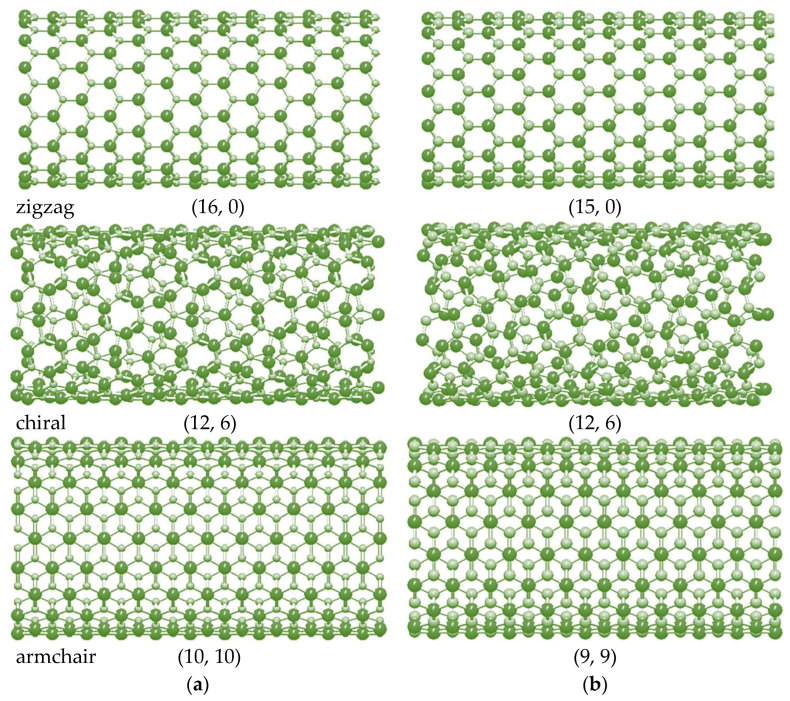
Configurations of (**a**) (16, 0) zigzag, (12, 6) chiral and (10, 10) armchair SWGeCNTs, and (**b**) (15, 0) zigzag, (12, 6) chiral and (9, 9) armchair SWSnCNTs, obtained with help of the software Nanotube Modeler© (version 1.8.0, ©JCrystalSoft). Ge and Sn atoms are shown in bright green; C atoms in pale green.

Figure 2 shows the schematic representation of non-chiral (zigzag and armchair) and chiral SWGeCNTs and SWSnCNTs with comparable diameters, $D_{n}$.

### 2.2. Numerical Modeling of Elastic Properties of GeC and SnC Nanosheets and Nanotubes

#### 2.2.1. Input for FE Model of 1D and 2D GeC and SnC Nanostructures

The nanoscale continuum modelling approach (NCM), also known as the molecular structural mechanics (MSM) approach, was used in the current study. The NCM approach consists of replacing the Ge–C and Sn–C bonds of the GeC and SnC nanostructures, respectively, by equivalent beam elements, well described by elasticity theory. Li and Chou [28] proposed relationships between the tensile, E_b_A_b_, bending, E_b_I_b_, and torsional, G_b_J_b_, rigidities of beams with the length *l*, which make up the equivalent continuum structure, and the bond-stretching, k_r_, bond-bending, k_θ_, and torsional resistance, k_τ_, force field constants, characterizing the respective molecular structure:

(4) $$E_{b}A_{b}=lk_{r},\;E_{b}I_{b}=lk_{\theta },\;G_{b}J_{b}=lk_{\tau }$$

Since no values were reported for the bond-stretching, k_r_, and bond-bending, k_θ_, force field constants of GeC and SnC nanostructures, two recognised methods for calculating k_r_ and k_θ_ of diatomic nanostructures were used for this purpose. The first method is based on Universal Force Fields (UFF) [29]. The bond-stretching, k_r_, force field constant is evaluated in the UFF method making use of the generalization of Badger’s rules [30] by the following expression [29]:

(5) $$k_{r}=664.12\frac{Z_{1}^{*}Z_{2}^{*}}{a_{A14-C}^{3}},$$

where $Z_{1}^{*}$ and $Z_{2}^{*}$ are the effective charges of the Ge (Sn) and C atoms, and $a_{A14-C}$ is the length of the Ge–C (Sn–C) bond.

According to Rappé et al. [29] UFF predicts that the bond-bending constant, k_θ_, of a diatomic nanostructure depends on the three-body angles between bond pairs Ge(Sn)–C–Ge(Sn) and C–Ge(Sn)–C, and on the effective charges of the atoms Ge(Sn) and C. This results in two different values for the bond-bending constant, k_θ1_ and k_θ2_, which are related to the effective charges ($Z_{1,2}^{*}$) by the following expression:

(6) $$\frac{k_{\theta 1}}{k_{\theta 2}}=\frac{Z_{2}^{*2}}{Z_{1}^{*2}}.$$

Considering expression (6) and knowing the angle between neighbouring bonds in the planar hexagonal structure ϑ = 120°, the equation for the bond-bending constant, k_θ_, proposed in the UFF method, takes the following form:

(7) $$k_{\theta 1(2)}=830.15\frac{Z_{2(1)}^{*2}}{{\left(\sqrt{3}a_{A14-C}\right)}^{3}},$$

where $Z_{1}^{*}$ and $Z_{2}^{*}$ are the effective charges of the Ge (Sn) and C atoms, $a_{A14-C}$ is the length of the Ge–C (Sn–C) bond.

The other established method to calculate k_r_ and k_θ_ force field constants combines ab initio DFT calculations with the analytical expressions for the surface Young’s modulus, $E_{s}$, and the Poisson’s ratio, ν, originated from molecular mechanics (MM) [31]. To calculate the bond-bending force constant $k_{\theta 1\left(2\right)}$, in accordance with DFT + MM method, Equation (6) was considered. Thus, to derive $k_{r}$, $k_{\theta 1}$ and $k_{\theta 2}$ force constants of the GeC and SnC nanostructures the following expressions were used:

(8) $$k_{r}=\frac{9E_{s}}{\sqrt{3}\left(1\;-\;\nu \right)},$$

(9) $$k_{\theta 1\left(2\right)}=\frac{E_{s}a_{A14-C}^{2}}{\left(1+\frac{Z_{1\left(2\right)}^{*2}}{Z_{2\left(1\right)}^{*2}}\right)\sqrt{3}\left(1+3\nu \right)},$$

where $E_{s}$ and ν are the surface Young’s modulus and the Poisson’s ratio of the GeC (SnC) sheet; $Z_{1}^{*}$ and $Z_{2}^{*}$ are the effective charges of the Ge (Sn) and C atoms; $a_{A14-C}$ is the length of the Ge–C (Sn–C) bond.

Literature data, necessary to evaluate the values of the bond-stretching, $k_{r}$, and bond-bending, $k_{\theta 1}$ and $k_{\theta 2}$, force constants for 1D and 2D GeC and SnC nanostructures are given in Table 1.

The bond-stretching, $k_{r}$, and bond-bending, $k_{\theta 1}$ and $k_{\theta 2}$, force constants, calculated by two aforementioned methods, and the torsional resistance force constant, $k_{\tau }$, taken from the DREIDING force field [32], where the torsional properties of the diatomic nanostructure are evaluated based solely on the hybridization of atoms, for GeC and SnC nanostructures, are summarized in Table 2.

Table 3 contains the geometrical and elastic properties of the beams (the input values for the numerical simulation), calculated making use of the values of the force field constants, $k_{r}$, $k_{\theta 1}$ and $k_{\theta 2}$, $k_{\tau }$, from Table 2.

The diameter, d, Young’s modulus, $E_{b}$, and shear modulus, $G_{b}$, of the beam were calculated, using Equation (4) and taking into account that *l* = $a_{A14-C}$, the beam cross-section area, $A_{b}=\pi d^{2}/4$, moment of inertia, $I_{b}=\pi d^{4}/64$, and polar moment of inertia, $J_{b}=\pi d^{4}/32$, as follows:

(10) $$d=2\sqrt{\frac{2\left(k_{\theta 1}+k_{\theta 2}\right)}{k_{r}}},$$

(11) $$E_{b}=\frac{k_{r}^{2}l}{2\pi \left(k_{\theta 1\;}+{\;k}_{\theta 2}\right)},$$

(12) $$G_{b}=\frac{{k_{r}^{2}k}_{\tau }l}{2\pi {\left(k_{\theta 1}+k_{\theta 2}\right)}^{2}}.$$

The Poisson’s ratio of the beam was evaluated by the molecular mechanics (MM) relationship [31,33] as follows:

(13) $$\nu_{b}=\frac{k_{r}l^{2}-\;3\left(k_{\theta 1}+{\;k}_{\theta 2}\right)}{k_{r}l^{2}+9\left(k_{\theta 1}+{\;k}_{\theta 2}\right)}.$$

#### 2.2.2. Geometrical Characteristics of GeC and SnC Nanosheets and Single-Walled GeC and SnC Nanotubes

As shown by Sakharova et al. [34], for hexagonal indium nitride nanosheets (NSs), and by Tapia et al. [35], for boron nitride NSs, the Young’s modulus of the nanosheets is almost independent of the NS size. Thus, single-layered nanosheets of GeC and SnC with sizes 6.77 × 6.88 nm^2^ and 7.10 × 6.36 nm^2^, respectively, were chosen for finite element analysis (FEA). Regarding SWGeCNTs and SWSnCNTs, three main configurations of these nanotubes were used in the FEA (see Table 4): zigzag (θ = 0°), chiral (family with θ = 19.1° as comprising the biggest number of NTs) and armchair (θ = 30°). SWGeCNTs and SWSnCNTs of similar diameters were chosen. The aspect ratio between nanotube length, $L_{n}$, and diameter, $D_{n}$, was around 30, to ensure that the elastic response of NTs did not depend on $L_{n}$ [36].

#### 2.2.3. FEA and Determination Elastic Properties of GeC and SnC Nanosheets and Nanotubes

The finite-element meshes of the nanosheets and nanotubes of GeC and SnC, used in FEA, were constructed using the Nanotube Modeler© software (version 1.8.0, ©JCrystalSoft, http://www.jcrystal.com, 13 June 2023). This software generates the Program Database files, which were afterwards converted, through the InterfaceNanotubes.NM in-house application [36], to the appropriate format for the ABAQUS^®^ code (Abaqus 2020, Dassault Systèmes®). The Ge–C and Sn–C bonds of the germanium carbide and tin carbide nanostructures, respectively, were replaced by equivalent 2-node cubic beam elements with circular cross-sections (see Table 3).

**Figure 3:**
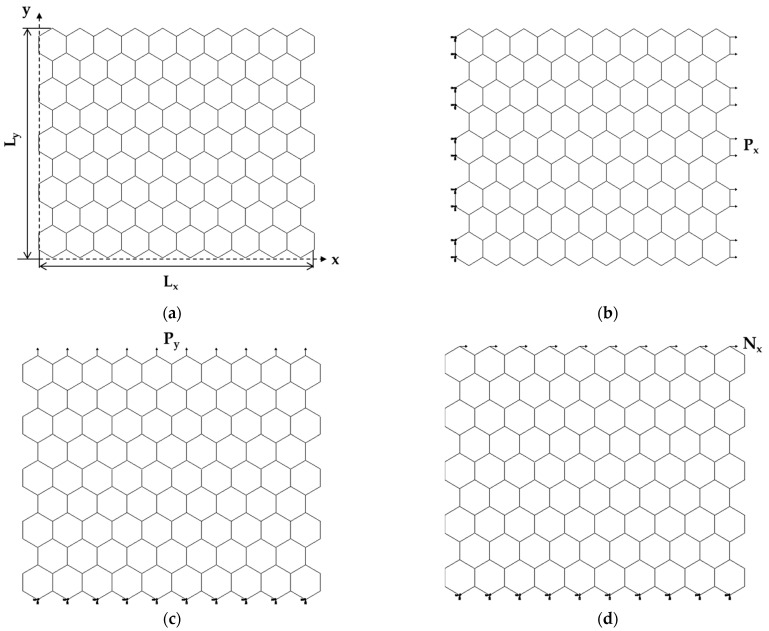
Schematic representation of: (**a**) geometrical parameters; (**b**) tensile loading in the horizontal direction (zigzag configuration), (**c**) tensile loading in the vertical direction (armchair configuration); (**d**) in-plane shear loading for the SnCNSs. The boundary conditions of the NSs are also shown.

The elastic response of the GeC and SnC nanosheets was studied under numerical tensile and in-plane shear tests, using the ABAQUS^®^ FE code. Figure 3 shows the geometry of the nanosheets (Figure 3a), and the boundary conditions of the three loading cases considered (Figure 3b–d).

In the first loading case, the nodes of the left edge of the NS were fixed, while an axial tensile force, P_x_, was applied to the opposite edge (Figure 3b). In the second case, the nodes of the lower edge of the NS were fixed, and an axial transverse force, P_y_, was applied at the opposite (upper) edge of the NS (Figure 3c). In the third loading case, the boundary conditions were the same as in the second case, and a shear force, N_x_, was applied to the upper-edge nodes of the nanosheet (Figure 3d).

**Figure 4:**
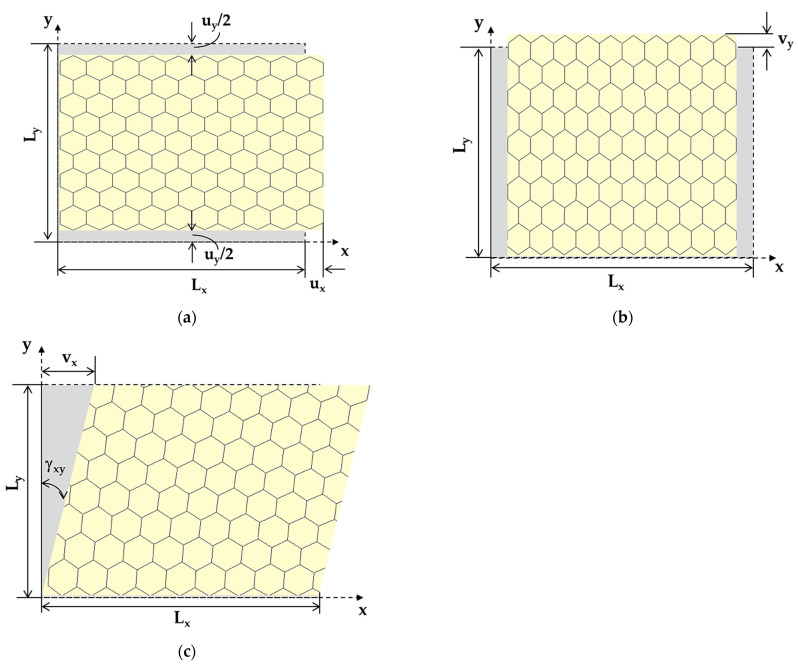
Schematic representation of deformed nanosheet for measuring: (**a**) NS axial displacement, $u_{x}$, under axial load P_x_, and NS transversal displacement, $u_{y}$, under axial load P_x_; (**b**) NS transversal displacement, $v_{y}$, under axial load, P_y_; (**c**) NS displacement along x-axis, $v_{x}$, under shear loading. The undeformed nanosheet is depicted in grey.

Under the applied force P_x_, the nanosheet elongated in the x direction and contracted in the y direction, which resulted in the axial displacement, $u_{x}$, and transversal displacement, $u_{y}$, respectively, as shown in Figure 4a. Consequently, the Young’s modulus along the x-axis, $E_{x}$, and the Poisson’s ratio, $\nu_{\mathrm{xy}}$, were evaluated by the following expressions, respectively [35]:

(14) $$E_{x}=\frac{P_{x}L_{x}}{u_{x}L_{y}t_{n}},$$

(15) $$\nu_{\mathrm{xy}}=\frac{u_{y}L_{x}}{{u_{x}L}_{y}},$$

where $L_{x}$ and $L_{y}$ are the NS side lengths (see Figure 3a); $t_{n}$ is the NS thickness; the transversal displacement, $u_{y}$, is measured at $x=L_{x}/2$ (see Figure 4a).

Given the lack of knowledge of the $t_{n}$ value, both for the GeCNSs and for the SnCNSs, instead of $E_{x}$, the surface Young’s modulus, $E_{\mathrm{sx}}$ (the product of the Young’s modulus by the NS thickness), was calculated as follows:

(16) $${E_{\mathrm{sx}}=E}_{x}t_{n}=\frac{P_{x}L_{x}}{{u_{x}L}_{y}}.$$

To calculate the Young’s modulus along the y-axis, $E_{y}$, the displacement of the NS in the y direction under the applied force P_y_, $v_{y}$, was taken from the FEA as shown in Figure 4b. Consequently, the surface Young’s modulus along y-axis, $E_{\mathrm{sy}}$, was assessed as follows:

(17) $$E_{\mathrm{sy}}=E_{y}t_{n}=\frac{P_{y}L_{y}}{v_{y}L_{x}}.$$

The surface shear modulus, $G_{\mathrm{sxy}}$ (the product of the shear modulus, $G_{\mathrm{xy}}$, by the NS thickness, $t_{n}$), of the GeCNSs and SnCNSs was calculated as follows [35]:

(18) $$G_{\mathrm{sxy}}=G_{\mathrm{xy}}t_{n}=\frac{N_{x}}{\gamma_{\mathrm{xy}}L_{x}},$$

where N_x_ is the in-plane shear force; $L_{x}$ is the bottom side length; $\gamma_{\mathrm{xy}}$ is the shear strain defined as:

(19) $$\gamma_{\mathrm{xy}}=\tan\frac{v_{x}}{L_{y}},$$

where $L_{y}$ is the lateral side length; $v_{x}$ is the displacement along the x-axis, as shown in Figure 4c; $v_{x}$ was measured in the central part of the nanosheet to avoid edge effects, where boundary and loading conditions were applied.

**Figure 5:**
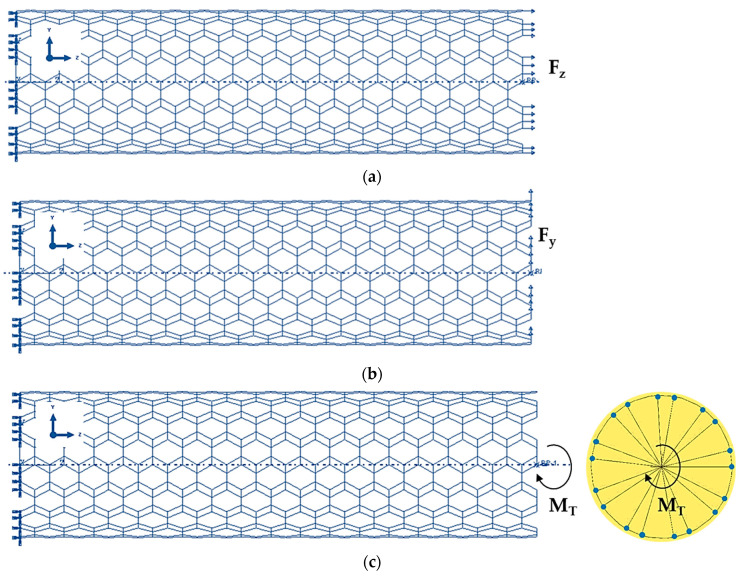
Boundary and loading conditions to test (9, 9) armchair SWGeCNTs in: (**a**) Tension; (**b**) Bending; (**c**) Torsion.

The ABAQUS^®^ FE code was also used to study the elastic response of SWGeCNTs and SWSnCNTs under tensile, bending, and torsion loading, as shown in Figure 5. The boundary conditions consisted in suppressing all degrees of freedom of the edge nodes of a side of the nanotube. The axial tensile force, $F_{z}$, the transverse force, $F_{y}$, and the torsional moment, M_T_, were applied to the opposite NT side, to carry out tensile (Figure 5a), bending (Figure 5b), and torsion (Figure 5c) tests. In this last test, the edge nodes were not allowed to move in the radial direction (see detail in Figure 5c).

In the FEA results of tensile, bending and torsion tests, the axial displacement, $w_{z}$, the transverse displacement, $w_{y}$, and the twist angle, ω, were evaluated. Therefore, the tensile, EA, bending, EI, and torsional, GJ, rigidities of the SWGeCNTs and SWSnCNTs with length $L_{n}$ were calculated using the following expressions:

(20) $$\mathrm{EA}=\frac{F_{z}L_{n}}{w_{z}},$$

(21) $$\mathrm{EI}=\frac{F_{y}L_{n}^{3}}{{3w}_{y}},$$

(22) $$\mathrm{GJ}=\frac{M_{T}L_{n}}{\omega }.$$

Equations (20)–(22) for EA, EI and GJ rigidities, were used to assess the Young’s, E, and shear, G, moduli, and the Poisson’s ratio of the SWGeCNTs and and SWSnCNTs, as follows [37,38]:

(23) $$E=\frac{\mathrm{EA}}{\pi t_{n}\sqrt{8\left(\frac{\mathrm{EI}}{\mathrm{EA}}\right)–t_{n}^{2}}},$$

(24) $$G=\frac{\mathrm{GJ}}{2\pi t_{n}\left(\frac{\mathrm{EI}}{\mathrm{EA}}\right)\sqrt{8\left(\frac{\mathrm{EI}}{\mathrm{EA}}\right)–t_{n}^{2}}},$$

(25) $$\nu =\frac{E}{2G}\;–\;1=\frac{\mathrm{EI}}{\mathrm{GJ}}\;–\;1,$$

where $t_{n}$ is the NT wall thickness, parameter identical to the NS thickness.

The calculation of the surface Young’s ($E_{s}=Et_{n}$) and shear ($G_{s}=Gt_{n}$) moduli allowed evaluation of the SWGeCNTs and SWSnCNTs elastic properties without the need to define a known $t_{n}$ value. Neglecting in Equations (23) and (24) the term $t_{n}^{2}$, since $t_{n}^{2}\ll 8\left(\frac{\mathrm{EI}}{\mathrm{EA}}\right)$, $E_{s}$, and $G_{s}$ of the SWGeCNTs and SWSnCNTs were assessed, respectively, by the following expressions:

(26) $$E_{s}=Et_{n}=\frac{\mathrm{EA}}{\pi \sqrt{8\left(\frac{\mathrm{EI}}{\mathrm{EA}}\right)}},$$

(27) $$G_{s}=Gt_{n}=\frac{\mathrm{GJ}}{2\pi \left(\frac{\mathrm{EI}}{\mathrm{EA}}\right)\sqrt{8\left(\frac{\mathrm{EI}}{\mathrm{EA}}\right)}}.$$

## 3. Results and Discussion

### 3.1. Elastic Properties of GeC and SnC Nanosheets

**Figure 6:**
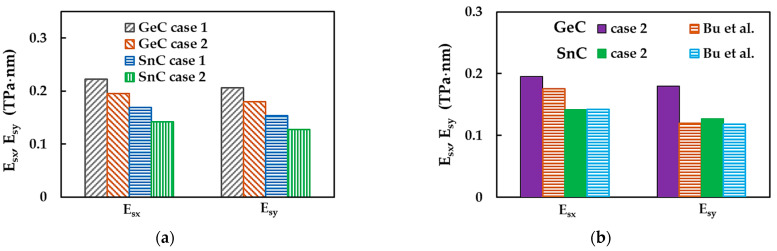
(**a**) Surface Young’s moduli, $E_{\mathrm{sx}}$ (zigzag) and $E_{\mathrm{sy}}$ (armchair), of GeCNS and SnCNS; (**b**) Comparison of the current surface Young’s moduli, $E_{\mathrm{sx}}$ and $E_{\mathrm{sy}}$, of GeCNS and SnCNS with those by Bu et al. [39] for 2D ph-GeC and ph-SnC nanostructures.

Figure 6a shows the surface Young’s moduli along zigzag, $E_{\mathrm{sx}}$ and armchair, $E_{\mathrm{sy}}$, directions, evaluated by Equations (16) and (17), respectively, for the nanosheets of GeC and SnC, considering case 1 and 2 of input parameters into numerical simulations (see Table 3). Figure 6b presents the $E_{\mathrm{sx}}$ and $E_{\mathrm{sy}}$ moduli of GeCNS and SnCNS, evaluated for case 2, plotted together with those obtained by Bu et al. [39] for 2D GeC and SnC nanostructures, whose lattice comprises hexagonal and pentagonal cells. In spite of differences in atomic arrangement, these penta-hexagonal (ph) germanium carbide and tin carbide NSs share some similarities, including mechanical properties, with GeCNSs and SnCNSs [39], which allows them to be chosen for comparison purpose. The surface Young’s modulus, $E_{\mathrm{sx},y}$, of GeCNS is at about 33% and 39% higher than $E_{\mathrm{sx},y}$ of SnCNS, for case 1 (UFF) and case 2 (DFT + MM), respectively (see Figure 6a). With regard to the effect of the input parameters on the surface Young’s modulus results, the difference between the $E_{\mathrm{sx},y}$ values calculated for case 1 (UFF) and case 2 (DFT + MM) is 14% and 20% for GeCNS and SnCNS, respectively. Very good agreement is observed when the current $E_{\mathrm{sx},y}$ moduli of SnCNS are compared with those evaluated using a Vienna ab initio simulation package (VASP) with DFT by Bu et al. [39] for ph-SnCNS (see Figure 6b). The Young’s modulus value, $E_{\mathrm{sx}}$, for GeCNS is in a reasonable concordance with that of ph-GeCNS [39], while the current value of $E_{\mathrm{sy}}$ is significantly higher than the respective Young’s modulus of ph-GeCNS.

The surface Young modulus for the zigzag NS configuration, $E_{\mathrm{sx}}$, is about 8% and 11% higher, for GeC and SnC nanosheets, respectively, than $E_{\mathrm{sy}}$ for the armchair NS configuration, whatever the case of the input parameters, 1 or 2. The ratios between the surface Young’s moduli for zigzag and armchair configurations are $E_{\mathrm{sx}}$/$E_{\mathrm{sy}}$ ≈ 1.08 and 1.11, for GeCNS and SnCNS, respectively. Thus, both germanium carbide and tin carbide NSs have anisotropic behaviour. For ph-GeC and ph-SnC nanosheets, these ratios are $E_{\mathrm{sx}}$/$E_{\mathrm{sy}}$ ≈ 1.47 and 1.20, respectively [39]. In fact, as reported in the work by Bu et al. [39], the 2D ph-GeC nanostructures were found to be considerably more sensitive to loading conditions than 2D ph-SnC. Moreover, the ratio $E_{\mathrm{sx}}$/$E_{\mathrm{sy}}$ ≈ 1.47 for ph-GeCNSs showed that they were characterized by greater anisotropy compared to the GeCNSs studied in the present work. This can explain the substantial difference observed between the current $E_{\mathrm{sy}}$ value for GeCNS and that evaluated for ph-GeCNS by Bu et al. [39].

**Figure 7:**
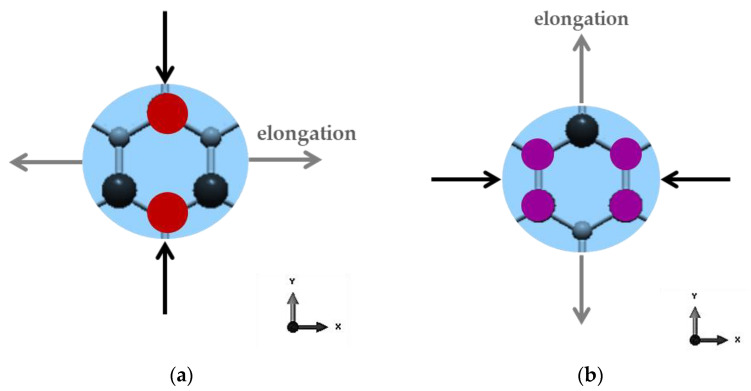
Exemplification of the elongation behaviour of hexagonal GeCNS and SnCNS lattices, under axial loading in (**a**) Zigzag and (**b**) Armchair directions.

The anisotropic behaviour of the GeC and SnC nanosheets can be explained by the dissimilar stresses necessary for elongation of the hexagonal NSs in the zigzag and armchair directions, under respective axial force, due to the arrangement of the atoms, as illustrated schematically in Figure 7.

**Figure 8:**
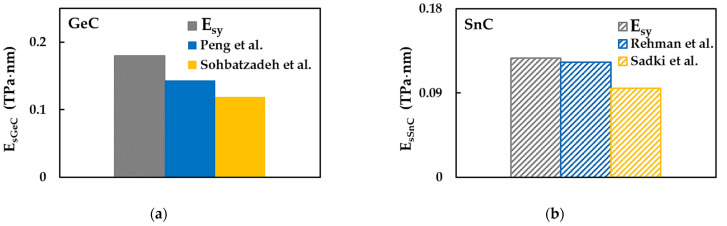
Comparison of the current surface Young’s modulus, $E_{\mathrm{sy}}$, calculated for case 2, for (**a**) GeCNS with those by Peng et al. [3] and Sohbatzadeh et al. [15]; (**b**) SnCNS with those by Rehnam et al. [13] and Sadki et al. [40].

The other existent works, to the best of our knowledge, report the surface Young’s modulus values regardless of the loading case, assuming that the GeC and SnC nanosheets are transversally isotropic [3,13,15,40]. Figure 8 shows the surface Young’s modulus, $E_{\mathrm{sy}}$, evaluated for GeCNS and SnCNS, considering case 2 of the input parameters, plotted together with results available in the literature.

Peng et al. [3] and Sohbatzadeh et al. [15] evaluated the surface Young’s moduli of GeCNSs, using VASP based on Kohn–Sham DFT and ab initio calculations within DFT, respectively. The values of $E_{\mathrm{sGeC}}$ reported in the works of Peng et al. [3] and Sohbatzadeh et al. [15] are nearly 20% and 34%, respectively, lower than the $E_{\mathrm{sy}}$ obtained in the current study (see Figure 8a). In turn, the difference between the surface Young’s moduli evaluated by Peng et al. [3] and by Sohbatzadeh et al. [15] is approximately 20%. The surface Young’s modulus of SnCNSs was reported in the works by Rehnam et al. [13] and Sadki et al. [40]. A good agreement (difference of 3.2%) is observed when the value of $E_{\mathrm{sSnC}}$ evaluated by Rehnam et al. [13], who employed VASP based on DFT for this purpose, is compared with the $E_{\mathrm{sy}}$ value obtained in the present study for case 2 of the input parameters (see Figure 8b). Sadki et al. [40] reported $E_{\mathrm{sSnC}}$ of about 34% lower than current $E_{\mathrm{sy}}$, making use of the Quantum Espresso (QE) ab initio simulation package within the pseudopotential approximation.

In view of the lack of previous results available, the current $E_{\mathrm{sGeC}}$ and $E_{\mathrm{sSnC}}$ surface Young’s moduli contribute to the establishment of a benchmark for evaluating the elastic properties of GeCNSs and SnCNSs by numerical methods.

The surface Young’s modulus results shown in Figure 6 and Figure 8 are summarized in Table 5.

**Figure 9:**
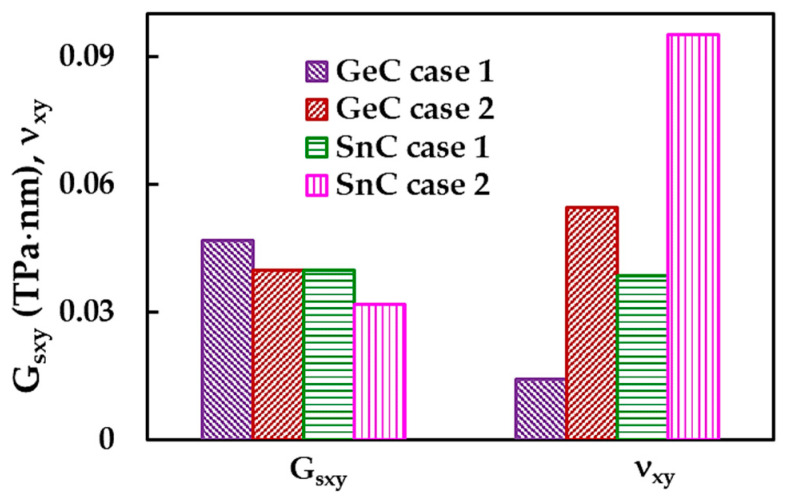
In-plane surface shear modulus, $G_{\mathrm{sxy}}$, and Poisson’s ratio, ν_xy_, of the GeCNS and SnCNS.

Figure 9 shows the in-plane surface shear modulus, $G_{\mathrm{sxy}}$, and in-plane Poisson’s ratio, ν_xy_, calculated using Equations (18) and (15), respectively, for GeCNS and SnCNS, in the two cases of input parameters. The surface shear modulus, $G_{\mathrm{sxy}}$, of the GeCNS is about 17.6% and 24.9% higher than $G_{\mathrm{sxy}}$ of the SnCNS, for case 1 (UFF) and case 2 (DFT + MM), respectively, whereas, the Poisson’s ratio, ν_xy_, calculated for the SnCNS is 2.7 and 1.7 times higher than that evaluated for GeCNS, for case 1 and 2, respectively.

Regarding the influence of the input parameters for the numerical simulation, a difference was found between the surface shear moduli, $G_{\mathrm{sxy}}$, evaluated for case 1 and for case 2, of 17.5% and 24.8% for the GeCNS and SnCNS, respectively. The value of ν_xy_ calculated for case 2 (DFT + MM) is about 4 and 2.5 times bigger than that for case 1 (UFF), for GeCNS and SnCNS, respectively.

The in-plane surface shear modulus and Poisson’s ratio results for GeCNS and SnCNS shown in Figure 9 are summarized in Table 6.

To complete the analysis of the elastic properties of the 2D germanium carbide and tin carbide nanostructures, their Young’s and shear moduli were compared with those of the 3D GeC and SnC counterparts. In the literature, the elastic moduli of 3D GeC and SnC compounds were reported by Hao et al. [41], Souadkia et al. [42], and Muthaiah and Garg [43]. Hao et al. [41] used first-principles DFT calculation to evaluate the shear and Young’s moduli of germanium carbide and tin carbide with a zinc blende-type structure. Souadkia et al. [42] also considered the zinc blende structure for GeC, while SnC was treated as being diamond-like, and they employed VASP based on of Density Functional Perturbation Theory (DFPT) to assess GeC and SnC shear and Young’s moduli. Muthaiah and Garg [43] used the QE package within the Voigt–Reuss–Hill approximation to calculate the elastic moduli of bulk hexagonal germanium carbide. Table 7 compares the Young’s and shear moduli of the 3D GeC and SnC compounds with those calculated in the current study for 2D GeC and SnC nanostructures. The value of the Young’s moduli of GeCNSs and SnCNSs is assessed as $E_{\mathrm{Ge}(\mathrm{Sn})C}=(E_{x}+E_{y})/2$, where $E_{x,y}={\;E}_{\mathrm{sx},y}/t_{n}$. Identically, the GeCNSs and SnCNSs shear moduli are calculated as $G_{\mathrm{Ge}(\mathrm{Sn})C}=G_{\mathrm{sxy}}/t_{n}$. In order to achieve a reliable comparison of the results, it is necessary to know the value of the nanosheet thickness, $t_{n}$. The $t_{n}$ values for the GeCNSs and SnCNSs, 0.380 nm and 0.381 nm, respectively, were taken from the work by Hess [44], where the monolayer thicknesses of compounds of the 14th group were approximated by the van der Waals (vdW) diameters.

The Young’s modulus reported in the literature for 3D GeC is in the range of 0.354–0.395 TPa, these values being about 1.5 and 1.3 times smaller than those evaluated for 2D GeC in the present study for case 1 and 2, respectively. In turn, the Young’s modulus values of the 3D SnC are nearly 1.8 and 1.5 smaller than those of the corresponding 2D compound, for case 1 and 2, respectively. Thus, the germanium carbide and tin carbide nanosheets have higher Young’s moduli than their bulk counterparts, which corresponds to the expectation of the superior mechanical characteristics of 2D nanostructures. On the contrary, the shear modulus of 3D germanium carbide and tin carbide compounds is higher (GeC) or equivalent (SnC) when compared with $G_{\mathrm{Ge}\left(\mathrm{Sn}\right)C}$ evaluated for GeCNSs and SnCNSs. This indicates that 2D structures have lower mechanical resistance to applied shear stress.

### 3.2. Elastic Properties of Single-Walled Germanuim Carbide and Tin Carbide NTs

#### 3.2.1. Rigidities of the SWGeCNTs and SWSnCNTs

**Figure 10:**
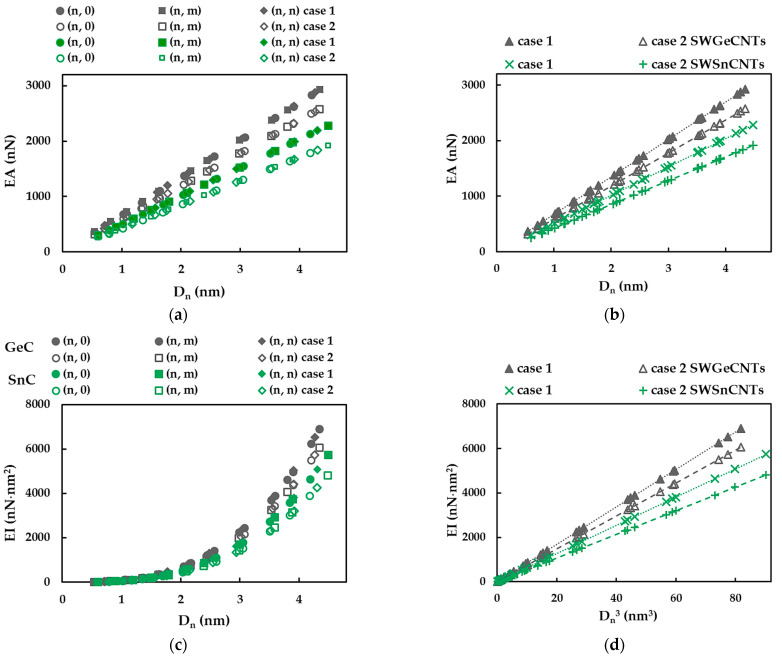

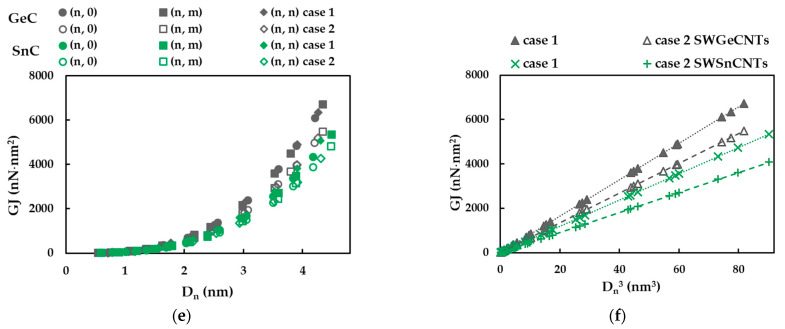
Evolutions of: (**a**,**b**) Tensile, EA, (**c**) Bending, EI, and (**e**) Torsional, GJ, rigidities as a function of the NT diameter, $D_{n}$, and (**d**) Bending, EI, and (**f**) Torsional, GJ, rigidities as a function of $D_{n}^{3}$, for the SWGeCNTs and SWSnCNTs from Table 4.

To analyse the tensile, EA, bending, EI, and torsional, GJ, rigidities of the SWGeCNTs and SWSnCNTs, the method used was proposed in the previous works by the authors [34,36,37,38,45,46]. This method consists in plotting the EA, EI and GJ rigidities, calculated by Equations (20)–(22), as a function of the nanotube diameter, $D_{n}$, as shown in Figure 10a,c,e, for cases 1 and 2 of the FE simulation input parameters (see Table 2). Each of the EA, EI and GJ rigidities follows the same trend with increasing nanotube diameter, regardless of the case of input parameters and the chiral angle (i.e., the NTs symmetry group). All three rigidities evaluated for case 1 (UFF) are greater than those for case 2 (DFT + MM), identically to the findings for indium nitride [34] and phosphide [45] NTs. It is worth noting that the EA, EI and GJ values obtained for the SWGeCNTs are higher than those for SWSnCNTs. In the same way as established for the NTs mentioned above [34,36,37,38,45,46], in the case of the SWGeCNTs and SWSnCNTs, the tensile rigidity, EA, follows a linear dependence with $D_{n}$ (Figure 10b), while the bending, EI, and torsional, GJ, rigidities follow a linear dependence with $D_{n}^{3}$ (Figure 10d,f).

**Figure 11:**
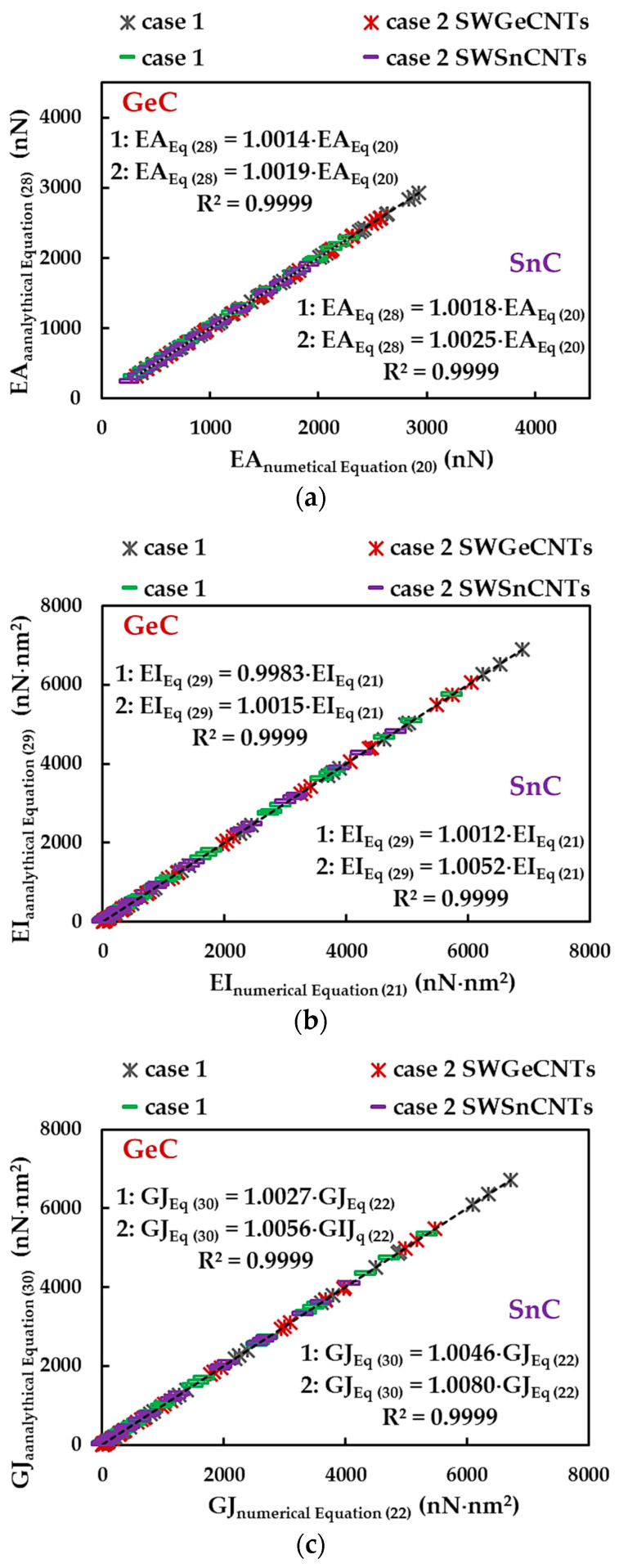
Comparison between (**a**) tensile, EA, (**b**) bending, EI, and (**c**) torsional, GJ, rigidities, acquired from FEA by Equations (20)–(22), and those calculated by the analytical expressions (28)–(30), considering case 1 and 2 of the input parameters.

Then, according to this method, the slops of the dash lines in Figure 10b,d,f were determined, according to the following expressions:

(28) $$\mathrm{EA}={\;\alpha }_{A14C}D_{n},$$

(29) $$\mathrm{EI}={\;\beta }_{A14C}{D_{n}}^{3},$$

(30) $$\mathrm{GJ}=\gamma_{A14C}{D_{n}}^{3},$$

where ${\;\alpha }_{A14C}$, ${\;\beta }_{A14C}$ and $\gamma_{A14C}$ are the fitting parameters and $D_{n}$ is the diameter of the SWGeCNTs and SWSnCNTs. The fitting parameters of Equations (28)–(30) are presented in Table 8. To analyse the accuracy of the abovementioned analytical expressions for assessment of the tensile, bending and torsional rigidities, the EA, EI and GJ rigidities calculated by Equations (28)–(30) were plotted versus those acquired from FEA, with help of Equations (20)–(22), in Figure 11. The trendline equations and the R-squared values shown in Figure 11 allow the conclusion that Equations (28)–(30) can be used for precise calculation of the three rigidities of the SWGeCNTs and SWSnCNTs.

**Figure 12:**
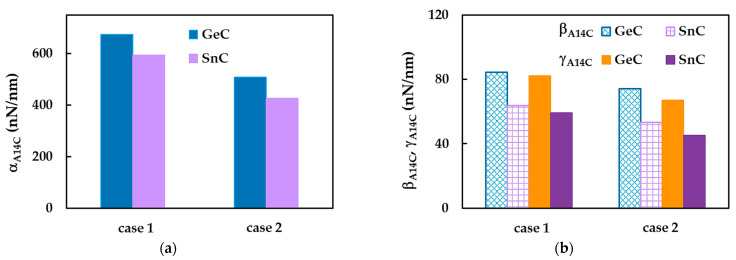
Fitting parameters (**a**) ${\;\alpha }_{A14C}$, (**b**) ${\;\beta }_{A14C}$ and $\gamma_{A14C}$ for SWGeCNTs and SwSnCNTs. Case 1 and 2 of the input parameters are considered.

Examining the ${\;\alpha }_{A14C}$, ${\;\beta }_{A14C}$ and $\gamma_{A14C}$ fitting parameters in Table 8, it is possible to quantify the ratios between the numerical tensile, EA, bending, EI, and torsional, GJ, rigidities of the SWGeCNTs and those values assessed for the SWSnCNTs, through the ratios $\alpha_{\mathrm{GeC}}/\alpha_{\mathrm{SnC}}$, $\beta_{\mathrm{GeC}}/\beta_{\mathrm{SnC}}$ and $\gamma_{\mathrm{GeC}}/\gamma_{\mathrm{SnC}}$, respectively. For case 1 of the input parameters, the results are $\alpha_{\mathrm{GeC}}/\alpha_{\mathrm{SnC}}$ ≈ 1.32, $\beta_{\mathrm{GeC}}/\beta_{\mathrm{SnC}}$ ≈ 1.32 and $\gamma_{\mathrm{GeC}}/\gamma_{\mathrm{SnC}}$ = 1.39, and for case 2 they are approximately 1.39, 1.39 and 1.48. The lower values of EA, EI and GJ rigidities, for the SWSnCNTs can possibly be explained by the longer length of the Sn–C bond, which is $a_{\mathrm{Sn}–C}$ = 0.205 nm, while the length of the Ge–C bond is $a_{\mathrm{Ge}–C}$ = 0.186 nm. With regard to the difference between the values of the fitting parameters obtained for case 1 (UFF) and those for case 2 (DFT + MM), the ratios $\alpha_{A14C}^{\mathrm{UFF}}/\alpha_{A14C}^{\mathrm{DFT}}$, $\beta_{A14C}^{\mathrm{UFF}}/\beta_{A14C}^{\mathrm{DFT}}$ and $\gamma_{A14C}^{\mathrm{UFF}}/\gamma_{A14C}^{\mathrm{DFT}}$ are 1.14, 1.14 and 1.23 for SWGeCNTs, and 1.19, 1.19 and 1.31 for SWSnCNTs. Thus, the input parameters calculated based on the UFF lead to higher tensile, bending and torsional rigidities than those evaluated with the input parameters derived from DFT results combining with MM relationships. The fitting parameters results from Table 8 are depicted in Figure 12, for easy comparison.

#### 3.2.2. Young’s Modulus of Single-Walled GeCNTs and SnCNTs

The surface Young’s modulus, $E_{s}$, of the SWGeCNTs and SWSnCNTs was evaluated with the aid of Equation (26). This equation uses the results of the numerical tensile and bending tests. Additionally, replacing in Equation (26) the tensile, EA, and bending, EI, rigidities by the expressions (28) and (29) and using the fitting parameters ${\;\alpha }_{A14C}$ and ${\;\beta }_{A14C}$, from Table 8, it is possible to obtain an analytical expression for $E_{s}$, which does not depend on $D_{n}$, as follows:

(31) $$E_{s}=\frac{\alpha_{A14C}}{\pi \sqrt{8\left(\frac{\beta_{A14C}}{\alpha_{A14C}}\right)}}.$$

**Figure 13:**
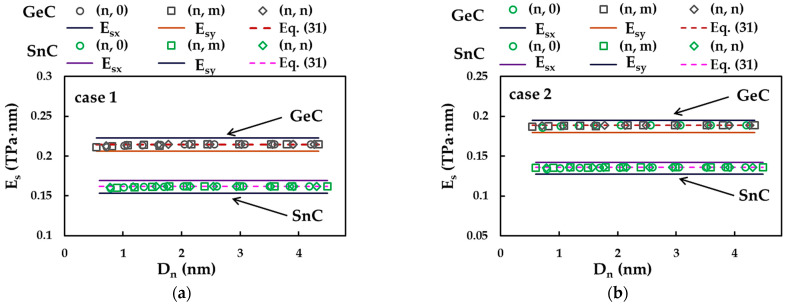
Evolutions of the surface Young’s modulus, $E_{s}$, for SWGeCNTs and SWSnCNTs as a function of the NT diameter, $D_{n}$, for cases (**a**) 1 and (**b**) 2 of input parameters. The surface Young’s moduli, $E_{\mathrm{sx}}$ and $E_{\mathrm{sy}}$, for GeCNS and SnCNS are also shown.

Figure 13 shows the evolutions of the $E_{s}$ value, calculated by Equation (26), with the diameter, $D_{n}$, of the SWGeCNTs and SWSnCNTs, for cases 1 and 2 of the input parameters. The surface Young’s moduli, $E_{\mathrm{sx}}$ (zigzag configuration) and $E_{\mathrm{sy}}$ (armchair configuration) evaluated for GeCNSs and SnCNSs, as well the values of $E_{s}$ assessed by Equation (31), are also plotted in Figure 13. For SWGeCNTs and SWSnCNTs, whatever the input parameter case, 1 or 2, as well the NTs type, whether non-chiral (zigzag and armchair) or chiral, the surface Young’s modulus is quasi-constant with the increase in the NT’s diameter, over the entire range of $D_{n}$ considered in the present study. In addition, the values of $E_{s}$ of SWGeCNTs and SWSnCNTs lie between $E_{\mathrm{sx}}$ obtained for zigzag configuration and $E_{\mathrm{sy}}$ for armchair configuration of the respective NS, tending towards the $E_{\mathrm{sx}}$ value and being slightly higher than the $E_{\mathrm{sy}}$ value.

The surface Young’s modulus results in Figure 13 are presented in Table 9. The mean differences between the $E_{s}$ values evaluated analytically by Equation (31) and those evaluated using FEA data with the help of Equation (26) are also shown in Table 8. As the largest average difference does not exceed 0.33%, it can be concluded that Equation (31) allows an accurate assessment of the SWGeCNTs and SWSnCNTs surface Young’s modulus, thus establishing a solid basis for evaluating the elastic properties of NTs without resorting to numerical simulation.

The $E_{s}$ value calculated for SWGeCNTs is 32% and 39% is greater than that obtained for SWSnCNTs in cases 1 and 2, respectively. As in the case of rigidities, such a difference can possibly be supported by the fact that the length of the Ge–C bond, $a_{\mathrm{Ge}–C}$ = 0.186 nm, is smaller than that of the Sn–C bond, $a_{\mathrm{Sn}–C}$ = 0.205 nm. The decreasing trend of the surface Young’s modulus with increasing bond length was also reported by Jiang and Guo [47] for single-walled SiC and diatomic NTs based on nitride and phosphide compounds, and by Sakharova et al. [45] for 13th-group phosphide NTs. Regarding the influence of the input parameters on the surface Young’s moduli results, the ratio $E_{s}^{\mathrm{UFF}}\;/{\;E}_{s}^{\mathrm{DFT}}$ between $E_{s}$ evaluated considering case 1 (UFF) and case 2 (DFT + MM) was approximately 1.41 and 1.19 for SWGeCNTs and SWSnCNTs, respectively, and is equal to those determined for corresponding 2D nanostructures, GeCNSs and SnCNSs (see Table 5).

**Figure 14:**
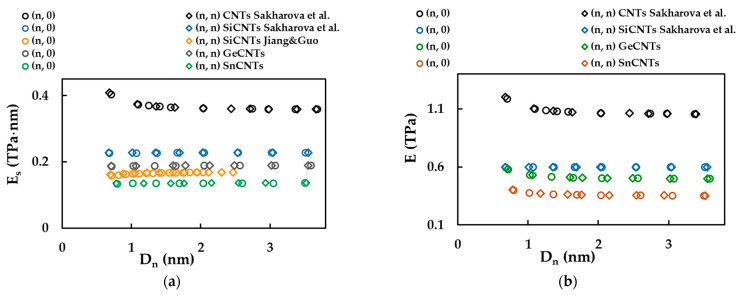
(**a**) Comparison of current surface Young’s modulus, $E_{s}$ , results for SWGeCNTs and SWSnCNTs with those for SWSiCNTs, by Jiang and Guo [47] and by Sakharova et al. [46], and for SWCNTs, by Sakharova et al. [36]; (**b**) Comparison of the Young’s modulus of SWGeCNTs and SWSnCNTs, calculated for the NT wall thickness, $t_{n}$ = 0.381 nm and 0.387 nm, respectively, with those of SWSiCNTs [46] and SWCNTs [36], for $t_{n}$ = 0.380 nm and 0.340 nm, respectively.

Direct comparison of $E_{s}$ for the SWGeCNTs and SWSnCNTs with the values by other authors is impeded by a lack of results in the literature. However, in the present study, the surface Young’s moduli of the SWGeCNTs and SWSnCNTs were compared with those evaluated for the SWSiCNTs [46] and SWCNTs [36], and the Young’s modulus of SWGeCNTs and SWSnCNTs with corresponding values for SWSiCNTs and SWCNTs, as shown in Figure 14a,b, respectively. The SWSiCNTs were selected for the purpose of comparison, as these NTs are representatives of the class of carbide nanotubes, together with SWGeCNTs and SWSnCNTs. As carbide NTs, in turn, are promising candidates to replace carbon nanotubes in numerous applications and nanodevices, this also motivated the consideration of SWCNTs for comparative study. To calculate the Young’s modulus, E, with the aid of Equation (23), it is necessary to know the value of the nanotube wall thickness, $t_{n}$. The values of $t_{n}$ = 0.381 nm and 0.387 nm, equal to the vdW diameter [44], were used for SWGeCNTs and SWSnCNTs, respectively. For the SWSiCNTs, $t_{n}$ = 0.380 nm from the same study [44] was chosen to calculate E from the results by Sakharova et al. [46].

It is worth noting that the present study shares the same modelling approach as the works of Sakharova et al. [36,46], chosen for comparative study. The current values of $E_{s}$ and E shown in Figure 14 were obtained considering case 2 of the input parameters. The surface Young’s modulus, $E_{s}$, for SWGeCNTs is 90% and 20% lower than the $E_{s}$ values evaluated for SWSiCNTs [46] and SWCNTs [36], respectively. The $E_{s}$ value of SWSnCNTs is 67% and 164% lower than that of SWSiCNTs [46] and SWCNTs [36], respectively. This should be considered for the correct design of nanodevices, for which GeC and SnC nanotubes are potential constituents.

**Figure 15:**
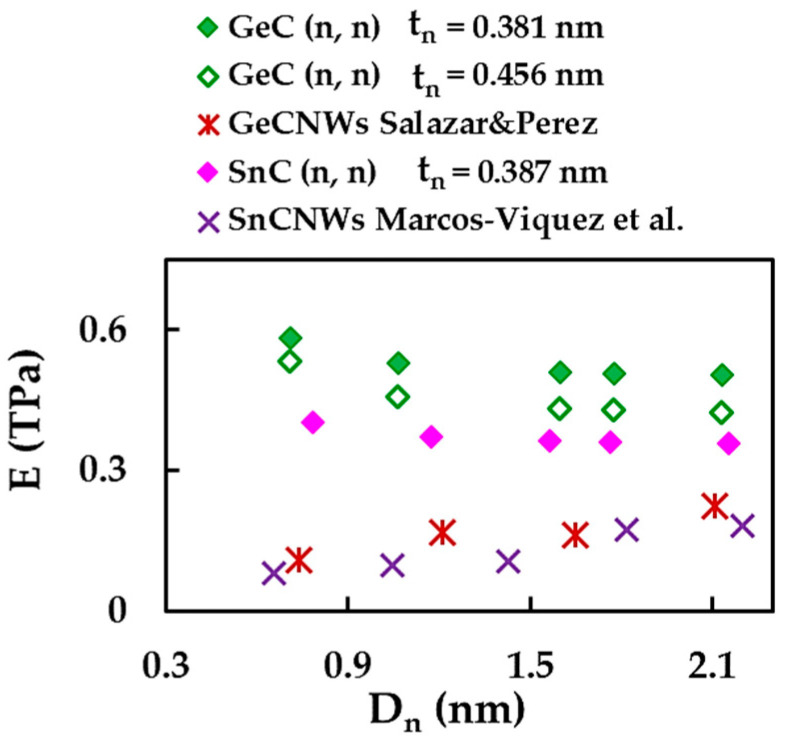
Comparison of the current Young’s modulus of the SWGeCNTs and SWSnCNTs with those of the GeCNWs and SnCNWs by Salazar and Pérez [48], and Marcos-Viquez et al. [49], respectively.

In conclusion, the current Young’s modulus results for SWGeCNTs and SWSnCNTs were compared with those reported for GeC and SnC nanowires (NWs) by Salazar and Pérez [48], and Marcos-Viquez et al. [49], respectively, as shown in Figure 15. Salazar and Pérez [48] evaluated the Young’s modulus of the GeCNWs with a diamond structure, implementing DFT within the local density approximation (LDA) in the SIESTA code. Marcos-Viquez et al. [49] employed the same modelling approach with the difference of using the general gradient approximation (GGA) instead of the LDA, to assess the Young’s modulus of the SnCNWs with a zinc blende structure. The values of E for the SWGeCNTs and SWSnCNTs were calculated by Equation (23), with $t_{n}$ = 0.381 nm and 0.387 nm, respectively. In addition, the SWGeCNTs Young’s modulus was assessed making use of $t_{n}$ = 0.456 nm, as reported by Song and Henry [27] for an interlayer distance of double-walled GeCNTs.

The values of E for the GeCNWs and SnCNWs are considerably lower than those obtained in the current study for SWGeCNTs and SWSnCNTs with comparable diameters. In fact, the hollow nanotube with hexagonal diatomic arrangement is able to attain higher elastic strain under uniaxial loading than the nanowire composed by the continuum lattice of the same binary compound.

#### 3.2.3. Shear Modulus and Poisson’s Ration of SWGeCNTs and SWSnCNTs

The surface shear modulus, $G_{s}$, of the SWGeCNTs and SWSnCNTs was evaluated by Equation (27), which resorts to the results of the numerical tensile, bending and torsional tests. Moreover, by replacing in Equation (27) the tensile, EA, bending, EI, and torsional, GJ, rigidities from expressions (28)–(30) and knowing the fitting parameters, ${\;\alpha }_{A14C}$, ${\;\beta }_{A14C}$ and ${\;\gamma }_{A14C}$, from Table 8, $G_{s}$ can be calculated analytically by the following expression:

(32) $$G_{s}=\frac{\gamma_{A14C}}{\pi \sqrt{32{\left(\frac{\beta_{A14C}}{\alpha_{A14C}}\right)}^{3}}},$$

which is independent of the NT’s diameter.

**Figure 16:**
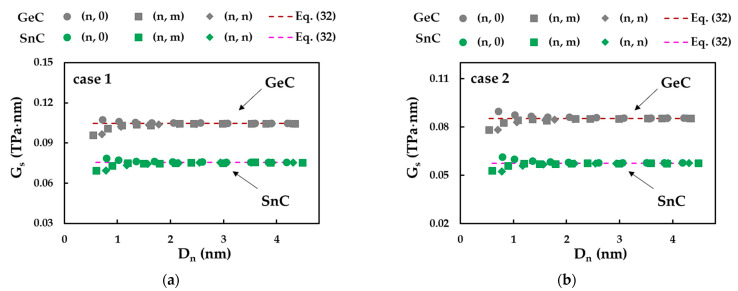
Evolutions of the surface shear modulus, $G_{s}$, for SWGeCNTs and SWSnCNTs as a function of the NT’s diameter, $D_{n}$, for cases (**a**) 1 and (**b**) 2 of input parameters.

Figure 16 shows the evolutions of the surface shear modulus, $G_{s}$, calculated by Equations (27) and (32), as a function of NT diameter, $D_{n}$, for SWGeCNTs and SWSnCNTs, considering cases 1 and 2 of the input parameters. For low $D_{n}$ values ($D_{n}$ < 1.76 nm) of SWGeCNTs and SWSnCNTs, $G_{s}$, decreases for (n, 0) NTs and increases for (n, m) and (n, n) NT, regardless of the case, 1 or 2. Then, the value of $G_{s}$ tends to a nearly constant value with increasing $D_{n}$, regardless of the chiral angle. These converged average values of $G_{s}$, shown in Table 10, are equal to those evaluated by Equation (32). As can be seen from Table 10, the greatest mean difference between the $G_{s}$ values obtained by the FEA and calculated analytically, does not exceed 0.09%. Thus, Equation (32) allows an accurate assessment of the surface shear modulus of SWGeCNTs and SWSnCNTs with diameter $D_{n}$ > 1.76 nm, establishing a solid basis for evaluation of the NTs elastic properties, without resorting to numerical simulation.

The $G_{S}$ value calculated for SWGeCNTs is 39% and 48% higher than that obtained for SWSnCNTs, for cases 1 and 2, respectively. The ratios $G_{s}^{\mathrm{UFF}}\;/{\;G}_{s}^{\mathrm{DFT}}$ ≈ 1.22 and 1.31 for SWGeCNTs and SWSnCNTs, respectively, indicate that the value of $G_{s}$ calculated for case 1 (UFF) is greater than for case 2 (DFT + MM). Both results are in line with those found for the surface Young’s modulus. The SWGeCNTs and SWSnCNTs shear moduli calculated by Equation (24), considering $t_{n}$ = 0.381 nm and 0.387 nm, respectively, are also shown in Table 10. The values of G obtained for the 1D GeC and SnC nanostructures are nearly 1.5 and 2 times greater than those for 3D GeC and SnC (see Table 7), respectively. Taking into account this result and the close values of the Young’s moduli obtained in the current study for the NSs and NTs of both binary compounds, it can be concluded that SWGeCNTs and SWSnCNTs have superior mechanical properties when compared with their bulk counterparts.

The Poisson’s ratio, ν, of the SWGeCNTs and SWSnCNTs was evaluated with the aid of Equation (25), making use of the EI and GJ rigidities, obtained from bending and torsional tests, respectively, and the ${\;\beta }_{A14C}$ and ${\;\gamma }_{A14C}$ fitting parameters of Table 8. This equation can be combined with expressions (29) and (30) for bending and torsional rigidities, in order to calculate ν independently of the NT diameter, as follows:

(33) $$\nu =\frac{\beta_{A14C}}{\gamma_{A14C}}-\;1.$$

**Figure 17:**
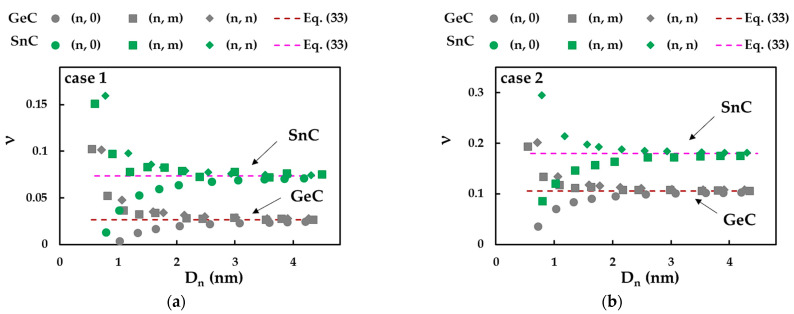
Evolutions of the Poisson’s ratio for SWGeCNTs and SWSnCNTs as a function of the NT’s diameter, $D_{n}$, for cases (**a**) 1 and (**b**) 2 of input parameters.

Figure 17 presents the evolutions of the Poisson’s ratio, evaluated by Equations (25) and (33), as a function of the NT diameter, $D_{n}$, for the SWGeCNTs and SWSnCNTs, in cases 1 and 2. For low $D_{n}$ values ($D_{n}$ < 2.75 nm) of SWGeCNTs and SWSnCNTs, ν decreases for (n, m) and (n, n) nanotubes and increases for (n, 0) nanotubes. Then, ν converges to the constant value determined by Equation (33), for all NTs, non-chiral and chiral, with $D_{n}$ > 2.75 nm. Thus, analytical expression (33) permits the accurate calculation of the Poisson’s ratio of SWGeCNTs and SWSnCNTs with diameters greater than 2.8 nm, as confirmed by the mean difference between the ν values calculated by this equation and those evaluated by FEA with aid of Equation (25) (see Table 11).

The value of ν for the SWSnCNTs is 2.8 and 1.7 times bigger than ν evaluated for the SWGeCNTs. In fact, the Poisson’s ratio decreases as the bond length increases, as was found in the works by Jiang and Guo [47], for nitride and phosphide NTs, and by Sakharova et al. [45], for phosphide NTs. With regard to the influence of the input parameters, the value of ν obtained for case 2 is 4.0 and 2.4 times bigger than that calculated for case 1, for SWGeCNTs and SWSnCNTs, respectively. Unlike the Young’s and shear moduli, whose values evaluated for case 1 (UFF) are greater than those for case 2 (DFT + MM), the Poisson’s ratio obtained using case 2 of the input parameters exceeds the ν value when case 1 is considered. This dissimilarity can be explained by the relationship between bending and torsional rigidities, EI/GJ, required to calculate ν by Equations (25) and (33). In fact, for case 1, the ratio $\beta_{A14C}/\gamma_{A14C}$ is approximately equal to 1.03 and 1.07, for the SWGeCNTs and SWSnCNTs, respectively, which means that EI and GJ rigidities are close to each other. For case 2, as $\beta_{A14C}/\gamma_{A14C}$ ≈ 1.11 and 1.18, for the SWGeCNTs and SWSnCNTs, respectively, the bending rigidity is greater than the torsional rigidity, resulting in a higher value of ν (see Equations (25) and (33)).

It is worth noting that the calculation of both the surface shear modulus and Poisson’s ratio makes use of the torsional rigidity, GJ (see Equations (27) and (25), respectively). This can probably explain the sensitivity of $G_{s}$ and ν to the nanotube chiral angle, θ, observed for the SWGeCNTs and SWSnCNTs with $D_{n}$ < 1.76 nm ($G_{s}$) and $D_{n}$ < 2.75 nm (ν). As shown previously for the SWBNNTs [36], the ratio $\mathrm{GJ}/D_{n}^{3}$ increases for (n, 0) zigzag NTs and decreases for (n, m) chiral and (n, n) armchair NTs, when the nanotube diameter is smaller than 1.5 nm. This behaviour is reflected in the evolutions of the surface shear modulus, $G_{s}$, of the SWGeCNTs and SWSnCNTs as a function of the NT’s diameter, $D_{n}$, as shown in Figure 16. Considering that the Poisson’s ratio is an inverse function of the torsional rigidity, the evolution of ν for $D_{n}$ < 2.75 nm is opposite, i.e., the value of ν decreases for (n, 0) NTs and increases for (n, m) and (n, n) NTs, as can be observed in Figure 15. On the other hand, it was shown that the ratio $\left(\mathrm{EI}/\mathrm{EA}\right)\left(1/D_{n}^{2}\right)$, for the non-chiral and chiral SWBNNTs, is nearly stable over the range of NT diameters, except for a slight increase observed for (n, m) and (n, n) NTs with $D_{n}$ < 0.9 nm [36]. As the Poisson’s ratio evaluated by Equation (25) does not depend on the tensile rigidity, EA, the scatter of the ν evolutions for SWGeCNTs and SWSnCNTs with diameters up to 2.75 nm is more pronounced than in the case of the evolutions of G, the value of which, assessed by Equation (24), depends on EA (see Figure 17).

To conclude the discussion, it should be noted that the input set computed with the aid of the UFF method (case 1) leads to higher values of the elastic constants, except of the Poisson’s ratio, compared to those evaluated by the DFT method combined with MM expressions (case 2). In fact, the $k_{r}$ and $k_{\theta }$ force field constants based on case 1 are greater than $k_{r}$ and $k_{\theta }$ used for calculating the input set for case 2 (see Table 2). The advantage of the UFF method is that only atom charges and bond length are required to calculate the bond-stretching and bond-bending force constants. The DFT + MM method, in addition to the atom charges and the bond length, makes use of the surface Young’s modulus and Poisson’s ratio of the nanostructure, data that are not always available and unambiguous.

## 4. Conclusions

The elastic properties of 1D and 2D graphene-like nanostructures of germanium carbide and tin carbide were assessed using numerical simulation based on the NCM/MSM approach. For the first time, systematic evaluation of the surface Young’s and shear moduli and Poisson’s ratio for GeC and SnC nanosheets (2D) and nanotubes (1D) was performed. The main conclusions are summarized below.

Two methods were used to calculate the force field constants, required to determine the input parameters in the numerical simulation. The sensitivity of the elastic properties of the 1D and 2D GeC and SnC nanostructures to the chosen set of input parameters was analysed. The elastic properties of GeC and SnC nanosheets and nanotubes, evaluated by numerical simulation with the input parameters based on the UFF calculation method, showed higher values than those evaluated using the input set of the DFT calculation. The only exception is the Poisson’s ratio of both GeC and SnC nanosheets and nanotubes.

The surface Young’s and shear moduli of GeCNSs, as well the three rigidities and the surface Young’s and shear moduli of the SWGeCNTs are superior to those evaluated for the corresponding nanostructures of SnCNSs or SWSnCNTs. On the contrary, the Poisson’s ratios of the GeCNSs and SWGeCNTs are smaller when compared to the respective values of SnCNSs and SWSnCNTs. These dissimilarities were attributed to the fact the length of the Ge–C bond is smaller than that of the Sn–C bond.

The previously established method, which makes use of the analytical expressions (28)–(30) to assess tensile, bending and torsional rigidities of NTs, was extended to two more cases of carbide nanotubes, GeCNTs and SnCNTs. The fitting parameters of the abovementioned equations, for these materials, were provided as a result of the present study.

The knowledge of these fitting parameters allows the calculation, without resorting to numerical simulation, of the surface Young’s modulus of single-walled GeC and SnC nanotubes comprising a wide range of diameters, and the surface shear modulus and Poisson’s ratio, limited to SWGeCNTs and SWSnCNTs of diameters bigger than 1.76 nm and 2.75 nm, respectively.

The 1D (nanotube) and 2D (nanosheet) structures of germanium carbide and tin carbide, NTs and NSs, with a hexagonal graphene-like lattice, show superior mechanical characteristics when compared with their 3D counterparts. The only exception is the shear moduli of GeCNSs and SnCNSs, whose values are lower than those of bulk GeC and SnC compounds. The obtained results establish a reference for the evaluation of the elastic properties of 1D and 2D graphene-like nanostructures of germanium carbide and tin carbide by theoretical methods.

## Figures and Tables

**Table 1:** Effective charges of atoms [29], and bond length, surface Young’s modulus and Poisson’s ratio, evaluated resorting to first-principles plane-wave calculations within DFT calculations for strain energy [1], in GeC and SnC nanostructures.

Compound	Atom 1	Atom 2	$Z_{1}^{*}$	$Z_{2}^{*}$	$a_{A14-C}$	E_s_, nN/nm [1]	ν [1]
			Charge [29]	Charge [29]			
GeC	Ge	C	2.789	1.912	0.186	142	0.33
SnC	Sn		2.961		0.205	98	0.41

**Table 2:** $k_{r}$, $k_{\theta }$ and $k_{\tau }$ force field constants for 1D and 2D germanium carbide and tin carbide nanostructures.

Compound	Case ^1^	$k_{r}$, nN/nm	$k_{\theta 1}$, nN nm/rad^2^	$k_{\theta 2}$, nN nm/rad^2^	$k_{\tau }$, nN nm/rad^2^
GeC	1	383	0.631	1.343	0.625
	2	367	0.456	0.970	
SnC	1	303	0.471	1.130	
	2	288	0.314	0.753	

^1^ Case 1 refers to the UFF calculation method and case 2 to DFT + MM.

**Table 3:** Geometrical and elastic properties of the beams utilized as input parameters in FE simulations of GeC and SnC nanosheets and nanotubes.

Compound	Case ^1^	*l*, nm [1]	d, nm Equation (10)	E_b_, GPa Equation (11)	G_b_, GPa Equation (12)	ν_b_ Equation (13)
GeC	1	0.186	0.2031	2196	695	0.24
	2		0.1762	2799	1227	0.33
SnC	1	0.205	0.2055	1875	732	0.29
	2		0.1722	2533	1485	0.41

^1^ Case 1 refers to the UFF calculation method, and case 2 to DFT + MM.

**Table 4:** Geometrical characteristics of the studied SWGeCNTs and SWSnCNTs.

NT Type	SWGeCNTs			SWSnCNTs		
	(n, m)	Diameter, $D_{n}$, nm	Length, $L_{n}$, nm	(n, m)	Diameter, $D_{n}$, nm	Length, $L_{n}$, nm
zigzag (n, 0), θ = 0	(7, 0)	0.718	22.13	(7, 0)	0.791	24.09
	(10, 0)	1.025	31.34	(9, 0)	1.017	30.55
	(13, 0)	1.333	39.13	(12, 0)	1.356	41.00
	(16, 0)	1.641	49.17	(15, 0)	1.695	50.23
	(20, 0)	2.051	60.88	(18, 0)	2.034	61.30
	(25, 0)	2.564	77.61	(23, 0)	2.600	77.90
	(30, 0)	3.076	92.67	(27, 0)	3.052	92.66
	(35, 0)	3.589	107.72	(31, 0)	3.504	105.57
	(38, 0)	3.897	117.76	(34, 0)	3.843	116.64
	(41, 0)	4.204	126.13	(37, 0)	4.182	125.87
chiral (n, m), θ = 19.1°	(4, 2)	0.543	16.91	(4, 2)	0.598	17.90
	(6, 3)	0.814	25.41	(6, 3)	0.897	26.81
	(8, 4)	1.085	33.25	(8, 4)	1.196	35.80
	(10, 5)	1.357	42.08	(10, 5)	1.495	46.25
	(12, 6)	1.628	49.52	(12, 6)	1.794	54.43
	(16, 8)	2.171	65.68	(14, 7)	2.093	64.15
	(18, 9)	2.442	74.52	(16, 8)	2.392	72.09
	(22, 11)	2.984	90.82	(20, 10)	2.990	90.22
	(26, 13)	3.527	106.97	(24, 12)	3.588	107.89
	(28, 14)	3.798	114.42	(26, 13)	3.887	117.88
	(32, 16)	4.341	130.57	(30, 15)	4.485	135.74
armchair (n, n), θ = 30°	(4, 4)	0.710	21.91	(4, 4)	0.783	23.97
	(6, 6)	1.066	33.02	(6, 6)	1.175	35.33
	(9, 9)	1.599	48.81	(8, 8)	1.566	48.61
	(10, 10)	1.776	54.61	(9, 9)	1.762	53.93
	(12, 12)	2.131	64.27	(11, 11)	2.153	64.58
	(14, 14)	2.487	75.87	(13, 13)	2.545	76.99
	(17, 17)	3.019	91.33	(15, 15)	2.936	89.41
	(20, 20)	3.552	106.80	(18, 18)	3.524	107.15
	(22, 22)	3.908	118.40	(20, 20)	3.915	117.80
	(24, 24)	4.263	128.06	(22, 22)	4.307	128.44

**Table 5:** Surface Young’s modulus results for GeCNSs and SnCNSs.

Reference	Compound	$E_{\mathrm{sx}}$, TPa nm	$E_{\mathrm{sy}}$, TPa nm	$E_{\mathrm{sx}}$ $/E_{\mathrm{sy}}$	$E_{\mathrm{sx}}^{\mathrm{UFF}}$ $/E_{\mathrm{sx}}^{\mathrm{DFT}}$	$E_{\mathrm{sy}}^{\mathrm{UFF}}$ $/E_{\mathrm{sy}}^{\mathrm{DFT}}$
Current study	GeC	0.223 ^1^	0.206 ^1^	1.080 ^1^	1.14	1.14
		0.195 ^2^	0.180 ^2^	1.086 ^2^		
	SnC	0.170 ^1^	0.154 ^1^	1.104 ^1^	1.19	1.19
		0.142 ^2^	0.127 ^2^	1.116 ^2^		
Bu et al. [39]	ph-GeC	0.176	0.120	1.470	–	–
	ph-SnC	0.142	0.118	1.200		
Peng et al. [3]	GeC	0.143		–	–	–
Sohbatzadeh et al. [15]		0.119				
Rehnam et al. [13]	SnC	0.123		–	–	–
Sadki et al. [40]		0.095		–	–	–

^1^ Case 1 refers to the UFF calculation method, and ^2^ case 2 to DFT + MM.

**Table 6:** Surface shear modulus and Poisson’s ratio results for GeCNSs and SnCNSs.

Compound	Case	$G_{\mathrm{sxy}}$, TPa nm	$\nu_{\mathrm{xy}}$	$G_{\mathrm{sxy}}^{\mathrm{UFF}}$ $/G_{\mathrm{sxy}}^{\mathrm{DFT}}$	$\nu_{\mathrm{xy}}^{\mathrm{DFT}}$ $/\nu_{\mathrm{xy}}^{\mathrm{UFF}}$
GeC	1	0.047	0.014	1.18	4.00
	2	0.040	0.055		
SnC	1	0.040	0.039	1.25	2.50
	2	0.032	0.095		

**Table 7:** Comparison of the Young’s and shear moduli results obtained for GeCNSs and SnCNSs with those for 3D GeC and SnC compounds in the literature.

Reference	Compound	$E_{\mathrm{Ge}\left(\mathrm{Sn}\right)C}$, TPa	$G_{\mathrm{Ge}\left(\mathrm{Sn}\right)C}$, TPa
current work	GeC (2D)	0.563 ^1^	0.123 ^1^
		0.492 ^2^	0.104 ^2^
	SnC (2D)	0.418 ^1^	0.103 ^1^
		0.348 ^2^	0.082 ^2^
Hao et al. [41]	GeC (3D)	0.354	0.152
	SnC (3D)	0.211	0.086
Souadkia et al. [42]	GeC (3D)	0.395	0.168
	SnC (3D)	0.257	0.104
Muthaiah and Garg [43]	GeC (3D)	0.389	0.169

^1^ Case 1 refers to the UFF calculation method and ^2^ case 2 to the DFT + MM approach used in the calculation of the input parameters for the FE simulation.

**Table 8:** Fitting parameters ${\;\alpha }_{A14C}$, ${\;\beta }_{A14C}$ and $\gamma_{A14C}$ for SWGeCNTs and SwSnCNTs.

Compound	Case	${\;\alpha }_{A14C}$, nN/nm	${\;\beta }_{A14C}$, nN/nm	$\gamma_{A14C}$, nN/nm	${\;\alpha }_{A14C}^{\mathrm{UFF}}/{\;\alpha }_{A14C}^{\mathrm{DFT}}$	$\beta_{A14C}^{\mathrm{UFF}}/\beta_{A14C}^{\mathrm{DFT}}$	$\gamma_{A14C}^{\mathrm{UFF}}/\gamma_{A14C}^{\mathrm{DFT}}$
GeC	1	674.39	84.25	82.08	1.14	1.14	1.23
	2	593.09	74.06	66.98			
SnC	1	509.02	63.60	59.24	1.19	1.19	1.31
	2	426.65	53.28	45.16			

**Table 9:** Surface Young’s modulus results for SWGeCTSs and SWSnCNTs.

Compound	Case	$E_{s}$, TPa nm	$E_{s}^{\mathrm{UFF}}$ $/E_{s}^{\mathrm{DFT}}$	Mean Difference, %
GeC	1	0.214	1.14	0.33
	2	0.189		0.25
SnC	1	0.162	1.19	0.27
	2	0.136		0.20

**Table 10:** Surface shear modulus results for SWGeCTSs and SWSnCNTs.

Compound	Case	^1^ $G_{s}$, TPa nm	G, TPa	$G_{s}^{\mathrm{UFF}}$ $/G_{s}^{\mathrm{DFT}}$	Mean Difference, %
GeC	1	0.104	0.273	1.22	0.01
	2	0.085	0.223		0.03
SnC	1	0.076	0.196	1.31	0.05
	2	0.058	0.150		0.09

^1^ Convergent average value.

**Table 11:** Poisson’s ratio results for SWGeCTSs and SWSnCNTs.

Compound	Case	^1^ ν	$\nu^{\mathrm{DFT}}/\nu^{\mathrm{UFF}}$	Mean Difference, %
GeC	1	0.026	4.0	0.20
	2	0.106		0.10
SnC	1	0.074	2.4	0.11
	2	0.180		0.04

^1^ Convergent average value.

## Data Availability

The data presented in this study are available on request from the corresponding author after obtaining permission from the authorized person.
